# A theoretical estimate for nucleotide sugar demand towards Chinese Hamster Ovary cellular glycosylation

**DOI:** 10.1038/srep28547

**Published:** 2016-06-27

**Authors:** Ioscani Jimenez del Val, Karen M. Polizzi, Cleo Kontoravdi

**Affiliations:** 1 School of Chemical & Bioprocess Engineering, University College Dublin, Belfield campus, Dublin 4, Ireland; 2 Centre for Process Systems Engineering, Department of Chemical Engineering, Imperial College London, South Kensington Campus, London SW7 2AZ, U.K; 3 Department of Life Sciences, Imperial College London, South Kensington Campus, London SW7 2AZ, U.K; 4 Centre for Synthetic Biology and Innovation, Department of Life Sciences, Imperial College London, South Kensington Campus, London SW7 2AZ, UK

## Abstract

Glycosylation greatly influences the safety and efficacy of many of the highest-selling recombinant therapeutic proteins (rTPs). In order to define optimal cell culture feeding strategies that control rTP glycosylation, it is necessary to know how nucleotide sugars (NSs) are consumed towards host cell and rTP glycosylation. Here, we present a theoretical framework that integrates the reported glycoproteome of CHO cells, the number of N-linked and O-GalNAc glycosylation sites on individual host cell proteins (HCPs), and the carbohydrate content of CHO glycosphingolipids to estimate the demand of NSs towards CHO cell glycosylation. We have identified the most abundant N-linked and O-GalNAc CHO glycoproteins, obtained the weighted frequency of N-linked and O-GalNAc glycosites across the CHO cell proteome, and have derived stoichiometric coefficients for NS consumption towards CHO cell glycosylation. By combining the obtained stoichiometric coefficients with previously reported data for specific growth and productivity of CHO cells, we observe that the demand of NSs towards glycosylation is significant and, thus, is required to better understand the burden of glycosylation on cellular metabolism. The estimated demand of NSs towards CHO cell glycosylation can be used to rationally design feeding strategies that ensure optimal and consistent rTP glycosylation.

Glycosylation is the most prevalent and impactful post-translational modification of recombinant therapeutic proteins (rTP)^1^,^2^. Twenty of the fifty highest selling pharmaceutical products in 2014 were glycoproteins and all twenty contain asparagine-linked (N-linked) complex carbohydrates (glycans), with two also containing serine/threonine-linked mucin type (O-GalNAc) glycans^3^. The relative abundance and monosaccharide composition of therapeutic protein glycoforms has been widely reported to influence the bioactivity, bioavailability, and biocompatibility of these molecules^1^,^2^. Given the close relationship between glycoform distribution and rTP safety and efficacy, understanding the fundamental aspects of protein glycosylation is of critical importance for the biopharmaceutical industry^1^,^2^,^4^.

Nucleotide sugars (NSs) are the direct link between cellular metabolism and the glycosylation process^4^,^5^. NSs are synthesised in the cytoplasm from nutrients commonly found in cell culture medium (e.g. glucose, glutamine, asparagine, and glycine) and are subsequently transported into the Golgi apparatus, where they are consumed in the glycosylation reactions. Several reports have linked decreased availability of NSs with reduced complexity of secreted glycans^6^,^7^,^8^. In addition, direct precursors for NS biosynthesis, such as N-acetylglucosamine (GlcNAc), galactose, N-acetylmannosamine (ManNAc), mannose, uridine, and cytosine, have been fed to the culture to ensure that NSs are sufficiently available to achieve adequate recombinant product glycosylation (reviewed recently in ref. 9). However, addition of these NS precursors has commonly been associated with reduced cell growth^10^,^11^,^12^,^13^; therefore, optimal NS precursor feeding strategies that can modulate recombinant product glycosylation while minimising the impact on cell growth are needed^12^,^14^. Alongside experimental work aimed at identifying such optimal feeding strategies, recently developed mathematical models^15^,^16^,^17^ could become a powerful tool for these purposes in the near future.

Crucially, the glycosylation of recombinant product (rTP), host cell proteins (HCPs), and lipids occurs simultaneously. This is substantiated by the close correlation observed between cell surface and rTP glycans reported recently^12^. As a consequence, a fraction of fed NS precursors does not reach their intended target, and identifying the demand of NSs required for HCP glycosylation is therefore necessary to define optimal NS precursor feeding strategies.

To date, two estimates for the demand of NSs towards mammalian cell HCP glycosylation have been made^16^,^17^. Both were obtained by combining the frequency of N-linked and O-GalNAc glycosylation sites reported across the entire SwissProt database^18^ with the relative abundance of each monosaccharide present in the reported glycome of human activated B-cells^16^ or CHO cells^17^. Despite providing useful initial approximations, these estimates have limitations given that they do not account for the relative abundance of each protein within the host organism’s proteome or the number of glycosylation sites on each protein. In addition, these estimates have not included the consumption of NSs towards glycolipid synthesis.

Here, we present a theoretical framework to estimate the demand of all nucleotide sugars consumed towards CHO HCP N-linked and O-GalNAc glycosylation, as well as for glycolipid synthesis. Our strategy integrates recent CHO proteomic^19^ and glycomic^20^,^21^ data to include (*i*) the relative abundance of all reported glycoproteins within the CHO proteome, (*ii*) the frequency of N-linked and O-GalNAc glycosylation sites across the proteome, and (*iii*) the monosaccharide composition of all N-linked and O-GalNAc HCP glycans. We also include the reported monosaccharide content of CHO lipids^22^, so that this sink for NSs is accounted for.

Our framework derives stoichiometric coefficients for the demand of all nucleotide sugars consumed for CHO HCP glycosylation and glycosphingolipid synthesis. The obtained stoichiometric coefficients are subsequently used to estimate how NS demand is partitioned between HCP, lipid, and rTP glycosylation^23^ in industrially-relevant mAb-producing CHO cells^24^,^25^,^26^. Our results indicate that NS consumption rates towards cellular and rTP glycosylation are within the same order of magnitude, suggesting that considering both is imperative to achieve a comprehensive description of NS metabolism.

The overall framework presented herein provides an approximation of NS demand towards CHO cellular glycosylation, which can aid in developing NS precursor feeding strategies that ensure control of rTP glycosylation without compromising cell growth and product yield. In addition, use of the estimated stoichiometric coefficients in advanced mathematical models for NS metabolism and glycosylation should improve model fidelity and take us one step closer to model-based design and control of feeding strategies that ensure optimal and consistent rTP glycosylation. Finally, we report the most abundant host cell N-linked and O-GalNAc glycoproteins in CHO cells as well as their individual contribution towards overall NS consumption. Monitoring the glycosylation profiles of this reduced set of glycoproteins could be used to track and predict the effect of NS precursor feeding strategies in the future.

## Materials and Methods

In order to obtain an improved estimate for the demand of NSs towards CHO cell glycosylation, we required an estimate of (*i*) the relative abundance of CHO HCPs, (*ii*) the weighted frequency of N-linked and O-GalNAc glycan sites per protein, (*iii*) the average glycoform distribution of each type, and (*iv*) NS demand for glycolipid synthesis. The workflow employed to obtain each of these components and the stoichiometric coefficients of NS consumption towards CHO HCP glycosylation are detailed in Fig. 1.

### Step 1: CHO HCP relative quantification

The reported proteome of CHO cells^19^ was compared with the SwissProt database^27^ using BLAST^28^ to obtain the closest reviewed homolog for each HCP. The sequence, length (*Len*_*i*_), and molecular weight (*MW*_*i*_) for each homolog were also retrieved from SwissProt. The obtained amino acid lengths and reported spectral counts^19^ (*SC*_*i*_) were then used to calculate the relative abundance (*Z*_*i*_) of each CHO HCP using Eqs 1 and 2, as described previously^29^,^30^. With the obtained values for *Z*_*i*_, the weighted average amino acid length (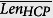) and molecular weight (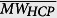) of CHO HCPs were calculated using Eqs 3 and 4. The list for all reported HCPs, the obtained homologs, and the calculations for *Z*_*i*_, 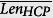, and 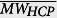 are presented in Supplementary Table ST1. The weighted relative abundance of the amino acids present in CHO HCPs 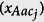 was obtained with Eq. 5, using the amino acid frequency in the sequence of each homologous protein (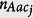) and *Z*_*i*_. The calculation of 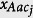 across the whole CHO proteome is presented in Supplementary Table ST2.


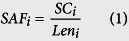



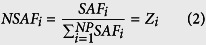



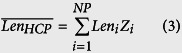



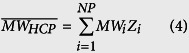



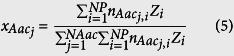


### Step 2: N-linked glycan sites per protein

The vast majority (>90%) of all reported N-linked glycosites in SwissProt have not been experimentally confirmed to be occupied^31^. In order to further refine the estimate by considering macroheterogeneity (variations in N-glycan occupancy of each potential site), the amino acid sequences for all potential N-linked glycoproteins were analysed using the NetNGlyc 1.0 server (http://www.cbs.dtu.dk/services/NetNGlyc/)^32^. To obtain a conservative estimate for N-linked glycosites, only ones found to be within the upper thresholds (++ and +++) were considered to be occupied. The obtained number of occupied N-linked glycosites (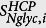) was multiplied by the respective protein’s relative abundance (*Z*_*i*_) and then summed to obtain the weighted number of N-linked glycosites across the entire CHO proteome (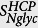), as shown in Eq. 6. All calculations performed to estimate 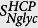, along with the contribution of each predicted N-glycoprotein to the weighted number of N-glycosites, are presented in Supplementary Table ST3.


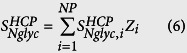


### Step 3: O-linked glycan sites per protein

As of February 2016, 2,375 O-GalNAc glycosylation sites were reported in a total of 182,101 eukaryotic glycoproteins in the SwissProt database^27^. However, a recent study by Yang and collaborators^21^ found 1,548 O-GalNAc glycosites in the proteome of CHO cells alone, suggesting that O-GalNAc glycosylation may be underreported in SwissProt. The low frequency of reported O-GalNAc sites is possibly due to the challenging analytical methods required for mapping, identifying, and quantifying these glycan types which lack a consensus amino acid sequon, may have core structure variability, and cluster densely in Ser/Pro/Thr-rich domains^33^.

In order to obtain the frequency of these post-translational modifications, the reported O-GalNAc glycoproteome of CHO cells was used^21^. The work of Yang *et al*. relied on engineering CHO cells to produce truncated O-GalNAc glycans that simplified glycopeptide enrichment using lectin chromatography and streamlined data analysis and interpretation for mapping all O-GalNAc glycosylation sites. The glycoproteome from this study was BLASTed^28^ against the SwissProt^27^ database to obtain a set of confirmed homologous O-GalNAc glycoproteins. This set was then aligned with the closest homologous proteins obtained for the entire CHO proteome. Once aligned, the number of O-GalNAc glycosites per HCP (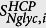) reported by Yang *et al*.^21^ was multiplied by the relative abundance of each HCP (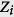) and summed (Eq. 7) to obtain the weighted average number of O-GalNAc glycosites across the CHO proteome (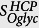). All calculations and the contribution of individual proteins to 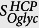 are presented in Supplementary Table ST4.


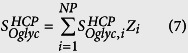


### Step 4. Weighted average monosaccharide composition of CHO HCP and mAb glycans

**Figure 2:**
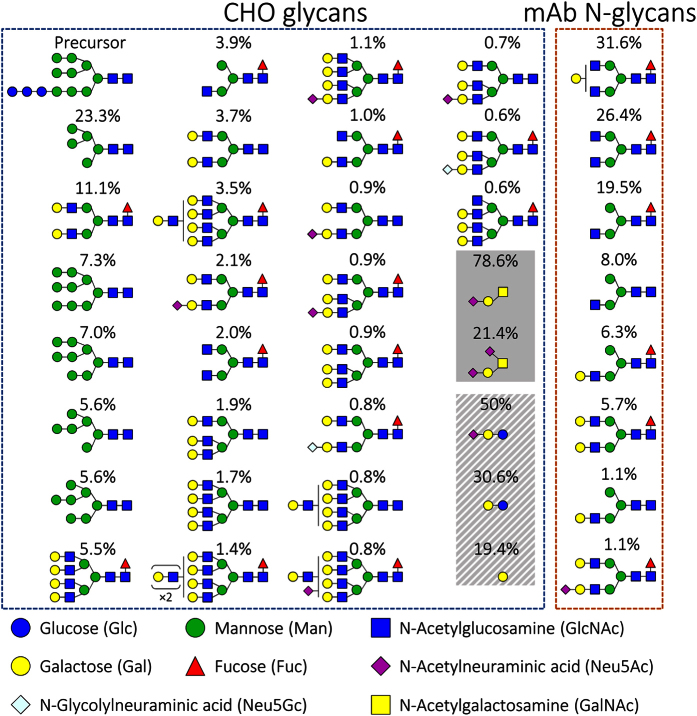
Glycan distributions produced by CHO cells. The distribution of N-linked and O-GalNAc glycans found on CHO HCPs is presented within the blue dashed box^20^. The O-GalNAc glycans are highlighted within the solid shaded box. The distribution of lipid-bound glycans is shown in the belted grey box^22^. The average composition of mAb Fc-bound N-linked glycans present on a commercial mAb (trastuzumab) produced by CHO cells^23^ is shown inside the orange dashed box.

In order to obtain the weighted average monosaccharide composition of CHO HCP glycans (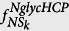
and 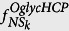), the reported^20^ relative abundance of each N-linked glycan species *l* and O-GalNAc glycan species *m* (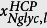 and 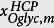, respectively) presented in Fig. 2 was multiplied by the frequency with which the monosaccharide species appears in each glycan (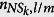), as shown in Eqs 8 and 9. Each monosaccharide present on the reported HCP glycans was assumed to be sourced from the respective nucleotide sugar (NS). All N-linked HCP glycans were assumed to contain three glucose (sourced from uridine diphosphate glucose – UDP-Glc) and nine mannose (sourced from guanosine diphosphate mannose – GDP-Man) residues, given that all N-linked glycans are synthesised from the Glc_3_Man_9_GlcNAc_2_ precursor oligosaccharide depicted on the top left corner of Fig. 2. These calculations are detailed in Supplementary Tables ST5 and ST6.

The weighted average monosaccharide composition of mAb glycans (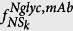) was obtained by combining the relative abundance of mAb constant fragment (Fc) N-glycans (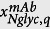) reported for trastuzumab^23^ (Fig. 2) with the frequency of each monosaccharide residue in these Fc carbohydrates (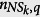), as shown in Eq. 10. The Fc glycoform distribution of trastuzumab^23^ was used because product glycan data is not reported in the work cited herein for the estimation of NS consumption between host cell and product glycosylation^24^,^25^,^26^. Furthermore, data for trastuzumab glycosylation was selected for the calculation of 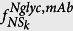 given that this product contains glycans that are representative of almost all commercially-available mAbs (complex biantennary with varying degrees of fucosylation and galactosylation^1^,^2^,^4^) and can be considered a ‘model’ CHO-derived product due to its isotype (IgG1 – the most common among therapeutic mAbs^4^) and the fact that it has been manufactured using CHO cells for nearly two decades^3^. Similarly to HCP glycans, all mAb Fc N-linked glycans were assumed to require three glucose and nine mannose residues for their formation. All calculations performed to obtain 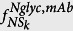 are presented in Supplementary Table ST7.


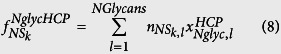



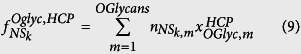



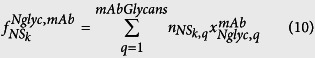


The monosaccharide content of total CHO cell lipids relative to total protein content (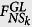) was obtained from the work by Briles and collaborators^22^. With these values, the distribution of CHO cell glycosphingolipids (Fig. 2) was calculated as shown in Supplementary Table ST8.

### Step 5: Stoichiometric coefficients of NSs per cell

The final step was to calculate the stoichiometric coefficients for the demand of NSs towards CHO HCP N-linked and O-GalNAc glycan synthesis (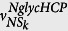 and 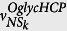, respectively), CHO cell glycolipid synthesis (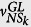), and mAb N-linked glycosylation (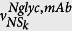). 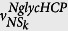, 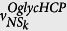, and 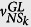 represent the weighted average number of nanomoles of NSs consumed towards each type of glycosylation per million cells. The stoichiometric coefficient of NS demand towards HCP N-linked glycosylation was obtained by combining the dry weight of CHO cells (*M*_*cell*_), the protein content of CHO cells (*Z*_*prot*_ in %w/w), the weighted average molecular weight of CHO HCPs (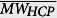), the weighted average number of N-linked glycosites across the CHO proteome (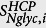), and the weighted average monosaccharide composition of CHO HCP glycans (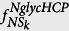), as shown in Eq. 11. The stoichiometric coefficient for NS demand towards CHO HCP O-GalNAc glycosylation was calculated in a similar fashion, as presented in Eq. 12. For these calculations, the dry weight of CHO cells reported by Carinhas *et al*.^34^ (*M*_*cell*_ = 271 *pg*/*cell*) and a cellular protein content of *Z*_*prot*_ = 74.2%^35^ were used. The value for dry cell weight was selected because it is representative of those that have previously been reported for CHO cells^36^,^37^, and *Z*_*prot*_ = 74.2% was selected given that similar values have been reported for several mammalian cell types, including CHO^34^,^35^,^36^,^37^,^38^. Although different assays have been used to quantify *Z*_*prot*_ (e.g. Bradford^34^ and Biuret^38^), the values that are reported typically range between 70% and 80%. The stoichiometric coefficient for NS consumption towards lipid glycosylation (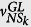) was obtained by multiplying the monosaccharide content of CHO cell lipids relative to total protein content (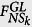)^22^ by the above mentioned values for *M*_*cell*_ and *Z*_*prot*_, as shown in Eq. 13. The calculations to obtain 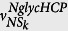, 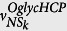, and 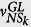 are shown in Supplementary Table ST9.

The stoichiometric coefficients for NS demand towards mAb Fc N-linked glycosylation 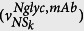 were calculated with Eq. 14 using the values of 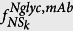 that were obtained as described in Step 4. The mAb was assumed to contain two N-linked glycosites on its Fc 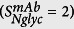. The molecular weight of the mAb was calculated using the amino acid sequence of trastuzumab and its weighted average glycan composition^23^ to yield a value of *MW*_*mAb*_ = 148,545 *g*/*mol*. The calculation of 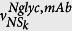 is presented in Supplementary Table ST9.


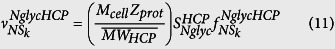



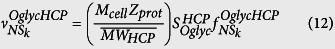



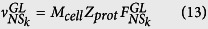



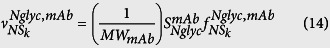


### Other types of cellular glycosylation

Although other types of glycosylation exist in mammalian cells, they have not been considered to greatly impact cellular NS demand due to their low abundance relative to the amount of HCP N-linked and O-GalNAc glycans and glycosphingolipids^39^,^40^. For example, O-mannose and O-fucose linked glycans have been indeed observed in the CHO cell glycoproteome, but at nearly undetectable levels^20^.

Similarly, dolichol pyrophosphate-linked oligosaccharides (Dol-PP-OS) have also been reported to be present in CHO cells^41^, but it is likely that NS demand towards this glycolipid pool is small compared to that of HCP and glycosphingolipid glycosylation. Intracellular accumulation of Dol-PP-OSs has been reported to depend on the availability of dolichol phosphate (Dol-P)^42^, which in turn, has been identified as a small fraction (only a few percent) of total cellular lipids in rat and mouse tissues^43^. In addition, the majority of monosaccharide constituents of the Dol-PP-OS precursor are cleaved off during glycan processing within the Golgi apparatus and are likely recycled for *de novo* synthesis of Dol-PP-OS. Therefore, it is possible to assume that NS consumption towards Dol-PP-OS synthesis is low compared to glycosphingolipid and HCP glycosylation and is, thus, negligible within our proposed framework.

### Proteoglycans

Proteoglycans, which contain linear carbohydrate chains constituted by tandem disaccharide motifs (glycosaminoglycans), are more commonly associated with cell adhesion in connective tissue^44^, and thus, may not be prevalent in CHO cells^39^. In order to evaluate whether this potential source of NS consumption is low compared to cellular N-linked, O-GalNAc, and glycosphingolipid glycosylation, the frequency of this glycosylation type was estimated based on the CHO proteome quantification described in Step 1 above.

Specifically, the database of closest confirmed homologs to the CHO proteome (ST1) was queried for presence of the forty-three known proteoglycans^44^. The weighted average frequency of glycosaminoglycan (GAG) sites per CHO HCP (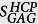) was obtained by multiplying the reported number of glycosaminoglycan (GAG) sites per proteoglycan 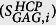 by the relative abundance of the corresponding protein (*Z*_*i*_) and summing this product across the entire CHO proteome, as shown in Eq. 15. These calculations are shown in Supplementary Table ST10.


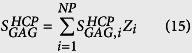


### Glycosylphosphatidylinositol (GPI) anchors

The glycophosphatidylinositol (GPI) anchor, which attaches proteins to the membrane of cells, also contains a carbohydrate component. The amount of free GPI and GPI-anchored proteins has also been reported to be low compared to N-linked, O-GalNAc, and glycosphingolipid glycosylation^39^,^40^. In order to estimate the abundance of GPI glycans in CHO cells, the number of potential GPI-anchored CHO HCPs (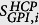) was obtained by submitting the amino acid sequences of all confirmed homologous CHO HCPs (ST1) to the PredGPI GPI-anchor prediction server (http://gpcr.biocomp.unibo.it/predgpi/pred.htm)^45^. The obtained 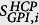 values were then multiplied by the relative abundance of each corresponding HCP (*Z*_*i*_) and summed (Eq. 16) to obtain the weighted average frequency of GPI-anchored HCPs (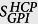) across the CHO proteome. These calculations are detailed in Supplementary Table ST10. The number of free GPI sites within CHO cells (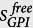) was obtained by multiplying 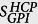 by the ratio of free to occupied GPI sites (3.7*oGPI*/*fGPI*) reported for CHO cells^39^, as shown in Eq. 17. These calculations are also presented in Supplementary Table ST10.


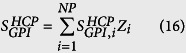



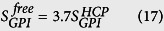


### Demand of NSs towards cellular and mAb glycosylation

Considering that the demand for NSs is partitioned between HCP, lipid, and mAb glycosylation, two limits can be identified: (*i*) when cell growth greatly exceeds mAb productivity and (*ii*) when mAb productivity considerably outweighs growth. Both scenarios could be observed in typical fed-batch culture, where during exponential growth phase, the demand of NSs towards cellular glycosylation would predominate, whereas during stationary phase, the demand of NSs towards cellular glycosylation would decrease considerably and demand towards mAb glycosylation would become significant. In order to cover both possibilities, data for the specific growth rate (*μ*_*g*_) and specific mAb productivity (*q*_*p*_) corresponding to a breadth of productivity to growth ratios 
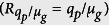
 reported for industrially-relevant CHO cells have been retrieved from recent publications^24^,^25^,^26^.

Specifically, two sets of values for *μ*_*g*_ and *q*_*p*_ were extracted from each reference, the first corresponding to a low productivity to growth rate ratio (Dool-L^24^, Chus-L^25^, and Kant-L^26^), and the second corresponding to a high productivity to growth ratio (Dool-H^24^, Chus-H^25^, and Kant-H^26^) (Table 1). All but the *μ*_*g*_ and *q*_*p*_ values obtained from Kant-H correspond to industrial CHO cell lines continuously expressing a recombinant mAb while growing exponentially. The selected *μ*_*g*_ and *q*_*p*_ values are considered typical for industrial CHO bioprocesses (*μ*_*g*_ between 0.03 h^−1^ and 0.05 h^−1^ and *q*_*p*_ between 20 pg/cell/day to 70 pg/cell/day)^46^,^47^. The values for Kant-H correspond to engineered CHO cells undergoing stationary growth while being cultured under mild hypothermic conditions and in the presence of sodium butyrate. The Kant-H values were selected to have an extremely high 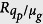 that would be representative of the limit for scenario (*ii*) described above (*q*_*p*_ = 94.05 pg/cell/day and *μ*_*g*_ = 0.011*h*^−1^). We must note that a *q*_*p*_ value of 100 pg/cell/day is commonly viewed as the upper threshold for CHO cell mAb specific productivity^48^. In all references, *q*_*p*_ was determined based on extracellular measurements of mAb titre. Chusainow *et al*. and Kantardjieff *et al*. determined mAb titre using ELISA, while Doolan *et al*. used protein A HPLC for mAb quantification.

In order to obtain estimates for NS demand towards HCP (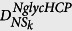 and 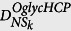), lipid (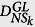), and mAb (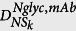) glycosylation under different growth and mAb productivity conditions, the *μ*_*g*_ and *q*_*p*_ values were simply multiplied by the stoichiometric coefficients calculated in Step 5, as shown in Eqs 18, 19, 20, 21. These calculations are detailed in Supplementary Table ST11.


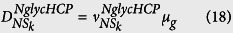



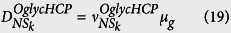



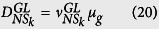



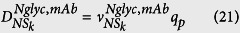


By combining the assumed values for *M*_*cell*_ = 271*pg*/*cell* 34 and *Z*_*prot*_ = 74.2%^35^ with the *μ*_*g*_ values obtained from literature^24^,^25^,^26^, cellular protein productivity can be estimated (Eq. 22). The obtained HCP, mAb, and total protein synthesis rates are shown in Table 1 and the corresponding calculations are presented in Supplementary Table ST11.


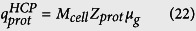


### Cellular glycosylation calculator

Although literature-derived experimental data has been used to perform all calculations reported herein, we must highlight that the supplementary file is a set of integrated spreadsheets that is amenable to analysis and modification by users to include experimental data for specific rTGP-producing CHO cell cultures. Users may readily adjust the relative abundance of HCP N-linked and O-GalNAc glycans (ST5 and ST6), the glycoform distribution of the recombinant product (ST7), the molecular weight and number of N-linked glycosites on the product (ST9), and the specific growth rate and productivity (ST11) to obtain stoichiometric coefficients for NS consumption towards recombinant product and cellular glycosylation for any system. These coefficients can, in turn, be used to estimate the partition of NS consumption towards cellular and rTGP glycosylation for user-defined CHO cell culture data and can also be used as inputs for models of CHO cell metabolism.

## Results and Discussion

### Relative abundance of CHO cell proteins

The most highly abundant individual proteins are those associated with the cytoskeleton (e.g. γ-actin, β-actin, β-tubulin, α-tubulin, vimentin), glycolysis (glyceraldehyde-3-phosphate dehydrogenase, fructose-bisphosphate aldolase A), chromatin (e.g. histones H4 and H2B), ribosomes (e.g. 40S ribosomal protein S12 and S3A), and those involved in protein transcription, translation, and folding (e.g. heat shock cognate 71 kDa protein, eukaryotic translation initiation factor 5A-1, elongation factor 1α-1, and peptidyl-prolyl cis-trans isomerase A). A full list of the CHO cell proteome including the relative abundance of each protein is presented in Supplementary Table ST1.

**Figure 3:**
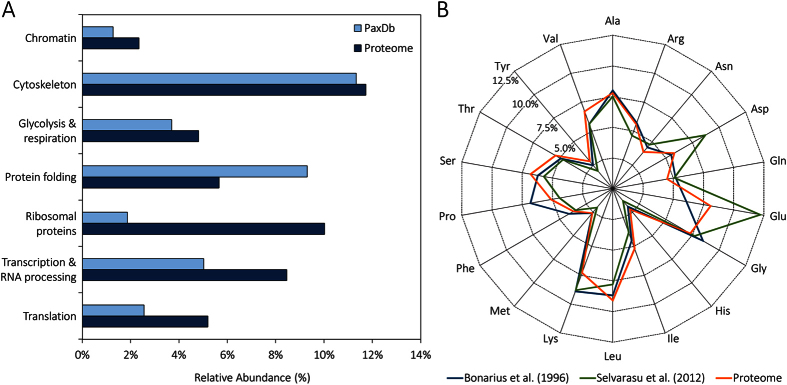
Relative abundance and amino acid composition of CHO host cell proteins. The relative abundance of the major categories for CHO HCPs are compared with those obtained from murine ovary cells^49^,^50^ (**A**). The amino acid composition of murine^38^ and CHO^51^ host cell proteins used in previous studies is compared with the one estimated from the CHO proteome (**B**).

Figure 3A compares the most abundant categories (as obtained through relative quantification and gene ontology analysis) of CHO HCPs with those obtained from protein relative abundance data of a murine ovary cell line (http://pax-db.org/)^49^,^50^. Both datasets are in relative agreement for cytoskeletal, chromatin, and glycolysis and respiration proteins. Large deviations can be observed for HCPs involved in protein transcription, translation, and folding. Specifically, HCPs involved in protein biosynthesis (transcriptional, translational, and ribosomal proteins) are in considerably higher abundance in CHO cells than in the murine ovary cell line. These differences are likely to be species-specific and are consistent with the phenotypic traits (rapid proliferation and high protein productivity) that characterise CHO cells.

In order to further validate the proteome-based quantification, the relative abundance of amino acids in the CHO HCPs was compared with previously reported data on murine^38^ and CHO^51^ HCP amino acid content (Fig. 3B). In general, the trends in amino acid content are similar across all datasets. When compared with the murine hybridoma data, the proteome-derived amino acid distribution diverges only slightly, with the highest observed deviations being for glutamate (+2.0%), proline (−1.7%), and lysine (+1.6%). These differences could be attributed to species-specific variations. When compared to the CHO dataset, the proteome-derived amino acid frequency diverges more. Particular differences can be seen for glutamate (−4.1%), aspartate (−2.9%), and lysine (−1.5%). As the major deviations with respect to the CHO dataset are observed for amino acids that are products of deamination, this result could be an artefact of the analytical method. Another possibility is that the analytical method used by Selvarasu *et al*.^51^ quantified total amino acid concentration (free amino acids plus those contained in protein). Because glutamate and aspartate are the key amino acids involved in the aspartate-malate shuttle, and CHO cells have high activity of this process^52^, a considerable amount of free glutamate and aspartate would be expected in this cell type. However, we cannot rule out that the deviation is due to the spectral counting quantification method employed.

### Frequency of N-linked and O-GalNAc glycosylation sites in CHO

The obtained frequencies for N-linked and O-GalNAc glycosylation sites in CHO HCPs are presented in Table 2. The frequency of O-GalNAc glycosylation is estimated to be 26.64 sites per 100 host cell proteins, while the frequency of N-linked glycosylation is lower, at 8.09 sites per 100 host cell proteins. These values are significantly lower than those derived in previous studies^16^,^17^, where N-glycosylation frequency was considered to be 170 sites per 100 host cell proteins and 158 O-GalNAc sites per 100 host cell proteins. These studies did not consider HCP relative abundance or the number of glycosylation sites per HCP. Precisely to refine these initial estimates, both of these components are included in our strategy to obtain more representative values of HCP N-linked and O-GalNAc glycosylation sites across the CHO proteome. When considering relative HCP abundance, only ~4% (mol/mol) of CHO HCPs contain N-glycosites and ~6.5% (mol/mol) contain O-GalNAc sites. Furthermore, a recent publication estimates the frequency of N-linked glycosylation sites across the *Pichia pastoris* proteome at 1 site for every 4,691 amino acid residues^53^. Using our estimated value for weighted average CHO HCP length (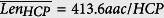) and N-glycosite frequency (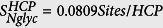) a value of 1 N-glycosite for every 5,102 amino acid residues is obtained (a deviation of only 8%). Although differences between *P. pastoris* and mammalian cell lines are expected, it would seem unusual for them to span two orders of magnitude. Irani *et al*.^53^ obtained their N-glycosite frequency value by dividing the previously reported number of N-linked glycosites in *P. pastoris* by the total number of amino acid residues in that organism’s proteome. Our strategy for obtaining O-GalNAc glycosylation site frequency is analogous, given that data for the O-GalNAc glycosylation sites of CHO cells was available^21^. Overall, we believe that the estimates presented herein and those reported by Irani *et al*.^53^ are more accurate than the previous ones because HCP relative abundance and glycosylation site frequency have been considered. The relative abundance of all N-linked and O-GalNAc glycoproteins, along with their weighted contribution to glycosylation site frequency are presented in Supplementary Tables ST3 and ST4, respectively.

**Figure 4:**
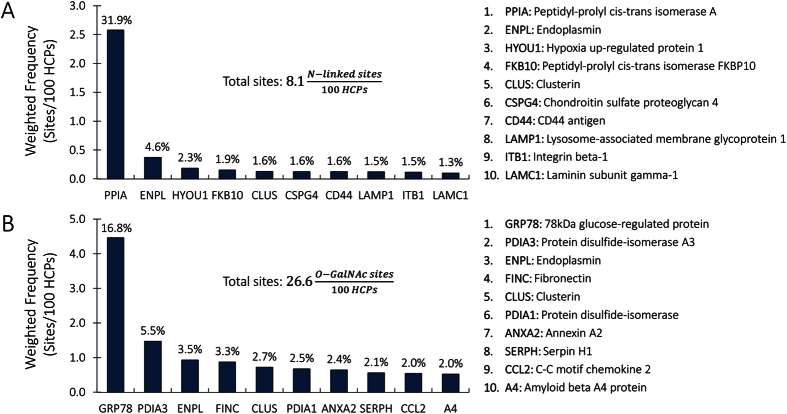
Major N-linked and O-GalNAc glycoproteins in CHO. The ten proteins that contribute most to total N-linked (**A**) and O-GalNAc (**B**) glycosites are shown. The labels above the bars show the percent contribution of each individual glycoprotein to the total number of N-linked and O-GalNAc glycosylation sites. The name of each glycoprotein is shown on the right.

An interesting feature of our estimates is that the ten highest contributors (out of a total of 437 predicted N-linked glycoproteins) account for 50% of the overall N-linked glycosite frequency. Similarly, the ten highest contributors to O-GalNAc site frequency (out of 346 predicted O-GalNAc glycoproteins) account for 43% of the total O-GalNAc glycosite frequency (Fig. 4). The fact that a small subset of proteins contributes so heavily to CHO HCP glycosylation could prove extremely useful for monitoring HCP glycosylation experimentally. If dynamic variations in abundance, site occupancy, and glycoform distribution of these top ten contributors is representative of the remaining host cell glycoproteins, they could be used as markers for dynamic variations in HCP glycosylation.

Nine of the eighteen most abundant glycoproteins in the CHO proteome are involved in protein folding (Fig. 4). Five of these are chaperones (endoplasmin, hypoxia up-regulated protein 1, clusterin, 78 kDa glucose-regulated protein, and serpin H1), while four are either disulphide isomerases (protein disulphide-isomerase A3 and protein disulphide isomerase) or peptide isomerases (peptidyl-prolyl cis-trans isomerase A, peptidyl-prolyl cis-trans isomerase FKB10). It would be interesting to evaluate if the carbohydrates bound to these proteins influence their activity. If so, we could envision a situation where HCP glycosylation is limited by NS availability, which, in turn, would lead to accumulation of unfolded or misfolded proteins in the endoplasmic reticulum. Furthermore, many of the enzymes involved in glycosylation are themselves glycoproteins^54^,^55^. Again, if activity of these enzymes relies on their carbohydrate composition, reduced NS availability could directly influence recombinant protein glycosylation while simultaneously decreasing glyco-enzyme activity, thus impacting overall protein glycosylation in a feed-forward manner. Although yet to be confirmed, these mechanisms could cause cascading effects whereby reduced NS availability may negatively impact protein folding as well as HCP and recombinant product glycosylation.

The remaining highly-abundant glycoproteins are cell adhesion and extracellular matrix components (chondroitin sulfate proteoglycan 4, laminin, fibronectin, annexin), cell surface receptors (CD44 antigen, integrin β), the C-C motif chemokine 2, the Lysosome-associated membrane glycoprotein 1, and the amyloid beta A4 protein. Given the role of carbohydrates in cell-cell interactions^56^, the high observed abundance of cell surface, extracellular matrix, and secreted glycoproteins is expected.

### Frequency of GAG, GPI, and O-Man glycosylation sites

#### GAG glycosites

Eleven proteoglycans were found in the CHO proteome, including syndecan, glypican, agrin, decorin, and lumican (ST10). These proteoglycans are reported to contain heparan, chondroitin, and keratin sulphate GAGs^44^. The total weighted frequency of proteoglycans across the CHO proteome was found to be 0.38 sites per 100 CHO HCPs (ST10). This value is over twenty-fold lower than the one obtained for N-linked glycosites and 70-fold lower than that of O-GalNAc glycosites. These results suggest that, although present, proteoglycans are in low abundance compared to N-linked and O-GalNAc glycoproteins, and do not contribute considerably to NS consumption towards CHO cellular glycosylation, an observation that has also been made experimentally^39^. We therefore believe that our assumption of disregarding these HCP glycosylation types as considerable sinks for NS consumption is valid.

#### GPI glycosites

The PredGPI GPI-anchor prediction server (http://gpcr.biocomp.unibo.it/predgpi/) identified 48 GPI-anchoring sites across the CHO proteome (ST10). The predicted GPI-anchoring sites were found on cell surface proteins (e.g. legumain, ephrin, the renin receptor, tissue plasminogen activator), secretory pathway proteins (e.g. ER-Golgi 24 kDa SNARE, transmembrane protein 115, Syntaxin-16), as well as various enzymes and chaperones (e.g. UMP-CMP kinase, lipoprotein lipase, tissue α-L-fucosidase, proteome assembly chaperone 2, peptidyl-prolyl cis-trans isomerase FKBP8). The weighted frequency of free and occupied GPI sites in CHO was found to be 0.447*sites*/100*HCP* and 1.655*sites*/100*HCP*, respectively. The total number of GPI sites (2.103*sites*/100*HCP*) is approximately four-fold lower than the frequency of HCP N-linked glycans and over 12-fold lower than HCP O-GalNAc glycosites. Given that the difference in glycosite frequency is relatively low, the demand of NSs towards GPI glycosylation was included in our estimates. The monosaccharide composition of GPI glycans was assumed to be the one reported for the Hamster prion protein GPI-anchor (Man_4_Neu5AcGalNAcGlcN)^57^. The stoichiometric coefficients of NS consumption towards GPI synthesis were calculated using the obtained GPI frequency and the monosaccharide distribution for the Hamster prion protein GPI anchor, as described for the N-linked and O-GalNAc glycans in the materials and methods section. The stoichiometric coefficients for lipid glycosylation presented in Table 2 are the sum of the stoichiometric coefficients for glycosphingolipid and GPI glycosylation. Individual values for GPI and glycosphingolipid stoichiometric coefficients are presented in Supplementary Table ST9.

#### O-Man glycosite frequency

Cadherins and plexins have been recently reported as the major O-mannosylated glycoproteins in humans^58^. This study reports 133 O-Man sites across 37 cadherins and 8 O-Man sites in 6 plexins out of a total of 235 identified O-Man glycosites. A third major source of O-Man glycosites was reported to be α-dystroglycan, with 13 sites. In order to evaluate whether O-mannose glycans are considerable sinks for NS consumption in CHO cells, the database for closes homologs to the CHO proteome (ST1) was queried for the presence of cadherins, plexins, and α-dystroglycan. Three cadherins (protocadherin fat1, protocadherin fat 3, and cadherin 9), four plexins (A4, C1, D1, and A1), and α-dystroglycan were found across the CHO proteome. The total relative abundance of the above seven proteins plus α-dystroglycan was found to be 0.014% of the CHO proteome. In total, these eight proteins contain 11 confirmed O-Man sites^58^. When this value is multiplied by the total relative abundance of these proteins, this yields a weighted O-Man glycosite frequency of 0.154 sites per 100 HCPs. This value is 50-fold lower than the number of N-linked glycosites and nearly 200-fold lower than that of O-GalNAc glycosites. This result confirms that O-Man glycans are in considerably lower abundance than other types of HCP glycosylation, and do not significantly contribute to NS consumption.

### Stoichiometric coefficients for NSs consumed towards CHO cell glycosylation

The stoichiometric coefficients range over three orders of magnitude (Table 2) and are higher for monosaccharides that are abundant in the average glycoform structure (e.g. GlcNAc in N-linked glycosylation) and lower for less abundant species (e.g. Neu5Gc). The high stoichiometric coefficients for GDP-Man and UDP-Glc towards N-linked glycosylation reflect the presence of their corresponding monosaccharides in the precursor oligosaccharide that initiates N-linked glycosylation, even though most of the mannose and all of the glucose residues are trimmed from the final structure through processing along the secretory pathway. It is worth noting that demand for these NSs may be lower considering that their monosaccharide components could be recycled after being cleaved off of the glycoprotein during the processing reactions.

Interestingly, the obtained stoichiometric coefficients for uridine diphosphate N-acetylglucosamine (UDP-GlcNAc) and uridine diphosphate N-acetylgalactosamine (UDP-GalNAc) towards HCP glycosylation are quite similar (1.248 *nmol*/10^6^*cells* and 1.160 *nmol*/10^6^*cells*, respectively) despite the notable difference in frequency of each glycosylation type. The difference in N-linked and O-GalNAc glycosite frequencies is offset by the high average GlcNAc content of HCP N-linked glycans (3.54 *nmol*_*GlcNAcl*_/*nmol*_*N−glycan*_) compared with that for O-GalNAc HCP glycosylation (1.0 *nmol*_*GlcNAcl*_/*nmol*_*O−glycan*_).

Only two NSs, uridine diphosphate galactose (UDP-Gal) and cytosine monophosphate N-acetylneuraminic acid (CMP-Neu5Ac), are consumed for all considered forms of glycosylation (N-linked and O-GalNAc HCP, lipid glycosylation, and mAb N-linked glycosylation). The highest obtained stoichiometric coefficient for UDP-Gal consumption corresponds to O-GalNAc HCP glycosylation (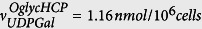) and is due to the high frequency of identified O-GalNAc glycosylation sites across the CHO proteome^21^ (Table 2) and the presence of galactose in all O-GalNAc glycans^20^ (Fig. 2). The stoichiometric coefficient of UDP-Gal towards lipid glycosylation was calculated as 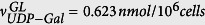, and surpasses that for HCP N-linked glycosylation (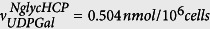). The higher stoichiometric coefficient for lipid glycosylation is likely due to the presence of galactose in the most abundant glycosphingolipids reported for CHO cells^22^,^59^.

Similarly to UDP-Gal, the highest obtained stoichiometric coefficient for CMP-Neu5Ac consumption is towards O-GalNAc HCP glycosylation (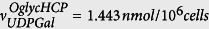). Again, this is due to the high frequency of O-GalNAc glycans across the CHO proteome and the high Neu5Ac content (at least one residue per glycan) of O-GalNAc glycans (Fig. 2). The second largest coefficient for CMP-Neu5Ac consumption is towards lipid glycosylation (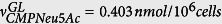), and is due to the presence of Neu5Ac on the G_M3_ ganglioside, which is the most abundant glycosphingolipid in CHO cells^22^,^59^. The low stoichiometric demand of CMP-Neu5Ac towards HCP N-linked glycosylation (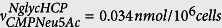) is due to the low abundance of the corresponding monosaccharide in CHO HCP N-linked glycans (Fig. 2).

### Partition of NS fluxes towards host cell and rTP glycosylation

#### Total NS fluxes

**Figure 5:**
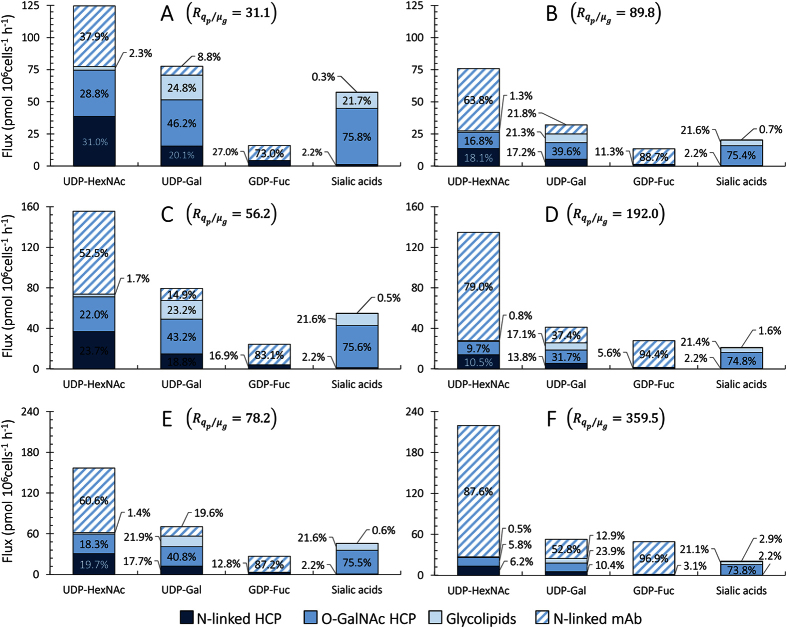
Distribution of nucleotide sugar fluxes towards cellular and mAb glycosylation. Theoretical distributions of NS fluxes towards N-linked HCP, O-GalNAc HCP, glycosphingolipids, and mAb N-linked glycosylation at low (**A,C,E**) and high (**B,D,F**) 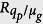 values. The distributions were estimated by multiplying the stoichiometric coefficient for each NS (Table 2), by values of *q*_*p*_ and *μ*_*g*_ reported for industrial cell lines (Table 1)^24^,^25^,^26^. The different datasets that are presented are Dool-L (**A**), Dool-H (**B**), Chus-L (**C**), Chus-H (**D**), Kant-L (**E**) and Kant-H (**F**). The percentage of total NS flux (axis) towards N-linked HCP (dark blue), O-GalNAc HCP (intermediate blue), lipid (light blue), and N-linked mAb (belted pattern) glycosylation is presented within the figure.

Total NS fluxes towards cellular and mAb glycosylation were estimated as described in the materials and methods section and Supplementary Table ST11. The highest demand of NSs towards cellular glycosylation can be observed at 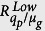, whereas the highest demand for product glycosylation will be observed at 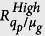, thus bounding a range of metabolic demand for cellular versus product glycosylation. The values for *q*_*p*_ and *μ*_*g*_ extracted from these publications were multiplied by the corresponding stoichiometric coefficients presented in Table 2 to obtain the percentage of NS fluxes directed towards cellular N-linked, O-GalNAc, and lipid glycosylation, as well as product N-linked glycosylation (Fig. 5). For analysis, the fluxes of UDP-GlcNAc and UDP-GalNAc towards cellular glycosylation have been summed (UDP-HexNAc), given that they share a common biosynthetic pathway where UDP-GalNAc is produced from the epimerization reaction of UDP-GlcNAc^16^.

Figure 5 shows that the flux of UDP-Gal and CMP-sialic acid towards glycosylation is highest at 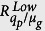. This trend is possibly due to the higher total protein productivity (HCP and mAb) observed under this regime (Table 1), which ranges from 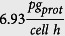 to 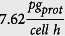 at 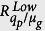, and is reduced to between 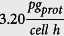 to 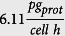 for 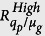. In contrast, more guanosine diphosphate fucose (GDP-Fuc) is consumed for 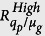 in the Chus and Kant datasets, while UDP-HexNAc is also consumed more for 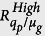 in the Kant dataset (Fig. 5). Specifically, the flux of GDP-Fuc towards glycosylation 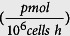 goes from 24.5 (Chus-L) to 28.1 (Chus-H) and from 27.2 (Kant-L) to 49.4 (Kant-H) (Fig. 5C–F). Similarly, the UDP-HexNAc flux increases from 157.2 (Kant-L) to 219.8 (Kant-H). For GDP-Fuc, this trend is likely due to the high percentage of protein productivity destined for mAb synthesis in the Chus-H and Kant-H datasets (48.8% and 64.1%, respectively), along with the considerably lower fucose content of HCP glycans 
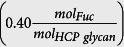
 compared to that of mAb glycans 
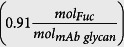
. Similar effects cause the observed increase in UDP-HexNAc flux towards glycosylation in the Kant-H scenario, although it may mainly occur due to the high *q*_*p*_, given that the HexNAc content 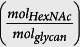 of HCP and mAb glycans is relatively similar (4.54 and 3.65, respectively).

### Flux distributions towards cellular and mAb glycosylation

#### GDP-Fuc and UDP-HexNAc flux towards HCP and mAb glycosylation

GDP-Fuc is exclusively consumed for N-linked glycosylation and its flux is distributed between host cell proteins and recombinant mAb according to demand. In each of the cases tested, the majority of GDP-Fuc flux goes towards mAb production (between 73% and 97%) due to the low abundance of fucosylated species in HCP glycans (Fig. 2). The flux of UDP-HexNAc is more evenly distributed between HCP and mAb glycosylation. Except for the Dool-L dataset, more than half of the UDP-HexNAc flux goes towards mAb glycosylation (Fig. 5), which is likely due to the high GlcNAc content of mAb N-glycans (Fig. 2). Despite the typically higher demand towards mAb glycosylation, the flux of UDP-HexNAc towards HCP glycosylation is significant and ranges between 11.9% and 59.8%. UDP-HexNAc consumption towards HCP glycosylation is almost evenly split between N-linked and O-GalNAc glycosylation. This arises from the similar stoichiometric coefficients obtained for UDP-GlcNAc towards HCP N-linked glycosylation and UDP-GalNAc towards HCP mucin-type glycosylation.

#### UDP-Gal and CMP-Neu5Ac flux towards cellular and mAb glycosylation

For all scenarios, HCP mucin-type glycosylation consumes the majority of CMP-Neu5Ac (between 73.8% and 75.8%), and the second highest demand for this NS is observed for lipid glycosylation (21.1% to 21.7%). Interestingly, the distribution of CMP-Neu5Ac consumption is almost independent of 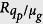, which is due to the scarcity of sialic acids in the mAb. For this reason, CMP-Neu5Ac consumption depends almost exclusively on the specific growth rate of CHO cells, and thus the distribution of its consumption remains nearly constant across different 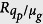 values.

The fraction of UDP-Gal flux consumed towards mucin-type glycosylation varies between 23.9% and 46.2% and is higher under conditions where 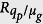 is low (and thus, antibody synthesis rate and utilisation of UDP-Gal for mAb glycosylation is low). Under 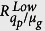, the UDP-Gal flux towards glycolipid synthesis is the next highest (21.9% to 24.8%), followed closely by HCP N-linked glycosylation (17.7% to 20.1%). Due to the low mAb productivity to growth ratio, only between 8.8% and 19.6% of the consumed UDP-Gal goes towards mAb N-linked glycosylation. We must note that UDP-Gal consumption towards mAb glycosylation surpasses HCP N-linked glycosylation in the Kant-L dataset, a result that is due to the relatively high 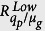 value observed for this engineered cell line.

Under the 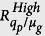 scenarios, consumption of UDP-Gal towards mAb glycosylation surpasses that of any other type of glycosylation, except for the Doolan-H dataset, where UDP-Gal consumption towards HCP O-GalNAc glycosylation is higher. Across all tested conditions for 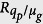, between 47.2% and 91.2% of UDP-Gal is consumed towards HCP N-linked, O-GalNAc, and lipid glycosylation, the only exception being the Kant-H dataset where 52.8% of all consumed UDP-Gal goes towards mAb glycosylation. In this context, we must highlight that the Kant-H data correspond to engineered CHO cells (overexpressing anti-apoptotic genes) being cultured under mild hypothermic conditions and in presence of sodium butyrate^26^. Under these conditions, the cells characterised by Kantardjieff *et al*. yield an exceptionally high specific mAb productivity (~100pg/cell/day), which causes the observed demand in UDP-Gal towards mAb glycosylation. Thus, it is possible to consider all other tested scenarios of 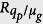 as more representative of typical CHO cell culture. In these typical CHO culture scenarios, more UDP-Gal is consumed towards cellular glycosylation than to product glycosylation, which is particularly relevant when considering NS precursor feeding strategies. Our results suggest that over half of NS precursors fed to control mAb galactosylation would be destined to cellular components and would not reach their intended target. This is consistent with previous findings where cell surface galactosylation was observed to increase more (up to four-fold) than mAb galactose content (up to two-fold) with different uridine-manganese-galactose feeding strategies^12^.

The changes in UDP-Gal flux distributions observed at varying 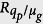 are the broadest observed in this study and are most likely due to the fact that this NS is the only one that is consumed for all considered forms of glycosylation (HCP N-linked and O-GalNAc, lipid, and mAb glycosylation). Given the observed variability of UDP-Gal flux distributions, it is no surprise that cell surface galactose content has been reported to correlate well with antibody galactosylation^12^. Furthermore, these results indicate that the partition of UDP-Gal between cellular and product glycosylation may be one of the underlying causes of galactosylation-associated microheterogeneity of mAbs produced in CHO: if a substantial proportion of available UDP-Gal is continuously consumed for cellular glycosylation, availability of this NS may fluctuate considerably throughout cell culture, and thus, lead to variations in rTP galactose content.

When considering that excess uridine and galactose supplementation have been reported to hinder cell growth and final product titre^10^,^12^,^13^, our results for UDP-Gal flux distributions further highlight the importance of having accurate estimates for NS consumption towards HCP glycosylation. Indicative estimates for the consumption of UDP-Gal towards HCP glycosylation are necessary to determine optimal uridine and galactose feeding strategies that ensure adequate rTP galactosylation while minimising (or completely avoiding) deleterious effects on cell growth and product yield.

The results obtained with our proposed framework are in qualitative agreement with previous findings, but the estimates could be influenced by: *i*) variations in the relative abundance of CHO HCPs and lipids, *ii*) variations in the occupancy of N-linked, O-GalNAc, and lipid glycosylation sites, and *iii*) changes in glycosylation microheterogeneity. All three of the above are likely to vary over time, depending on cell culture conditions, NS precursor supplementation strategy, growth phase, and cellular metabolic state. Indeed (and possibly due to a combination of proteomic, macroheterogeneity, and microheterogeneity effects), qualitative variations in CHO cell surface glycosylation have been reported^12^,^60^. The overall framework presented herein could be readily applied, via the CHO cellular glycosylation calculator provided in the supplementary file, to any experimental data so that variations in the demand for NSs towards cellular and rTGP glycosylation can be represented for different cell lines grown under different culture conditions. Experimentally, these limitations can also be addressed by monitoring the relative abundance and glycosylation of the subset of CHO proteins that were found to contribute most to HCP glycosylation.

## Conclusions

The work presented herein describes a strategy to estimate the metabolic demand of NSs towards protein and lipid glycosylation in CHO cells. The estimate combines the relative abundance of individual CHO host proteins with the number of N-linked and O-GalNAc glycosylation sites present on each HCP and the reported CHO HCP glycome. The stoichiometric demand of NSs towards glycolipid (glycosphingolipid and GPI anchor) synthesis has also been included. Overall, our results show that the consumption of NSs towards HCP glycosylation is, in most cases, significant and cannot be neglected when rationally designing NS precursor feeding strategies or while developing mechanistic mathematical models for cell growth and recombinant protein glycosylation. The obtained stoichiometric coefficients for NS consumption towards cellular glycosylation are a first approximation towards a fuller quantitative understanding of how the process of HCP, glycosphingolipid, and rTP glycosylation are integrated. In addition, we have identified a subset of CHO HCPs which, if monitored for abundance, glyco-site occupancy, and glycan microheterogeneity, could refine the obtained stoichiometric coefficients, and thus improve our quantitative understanding of cellular and rTP glycosylation. In the future, analysis of the overall burden of protein glycosylation on cellular metabolism will lead to mechanistically-defined feeding strategies for quantitative and optimal control of rTP glycosylation.

## Additional Information

**How to cite this article**: del Val, I. J. *et al*. A theoretical estimate for nucleotide sugar demand towards Chinese Hamster Ovary cellular glycosylation. *Sci. Rep.* **6**, 28547; doi: 10.1038/srep28547 (2016).

## Supplementary Material

Supplementary Tables ST1-ST11

## Figures and Tables

**Figure 1:**
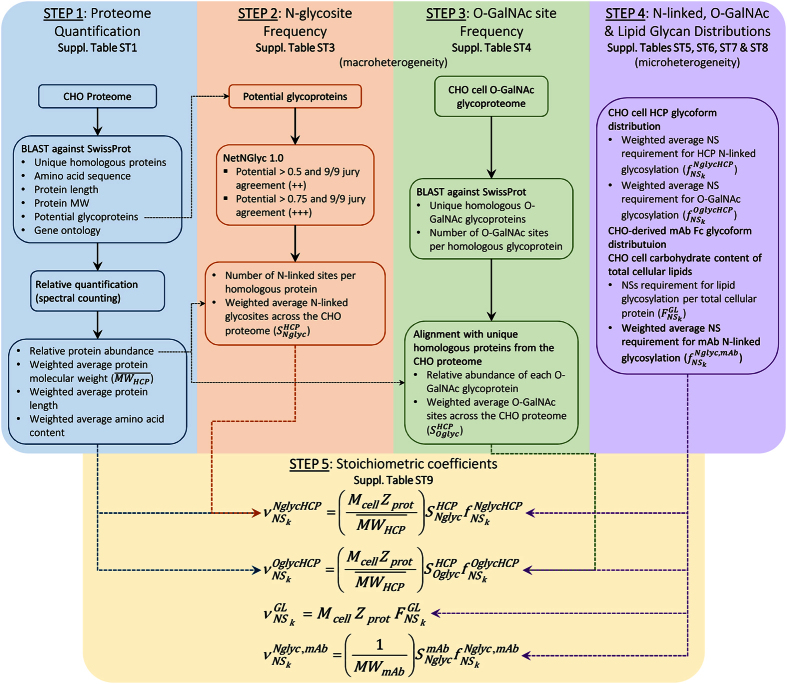
Workflow for the estimation of stoichiometric coefficients for nucleotide sugar demand towards CHO cell and mAb glycosylation. Step 1: the CHO proteome^19^ was BLASTed^28^ against the SwissProt database^27^ to obtain the corresponding confirmed homologous proteins. The length, amino acid sequence, molecular weight, and potential N-linked glycosylation sites for each homologous protein were extracted from the database. Using the protein length and the spectral counts available from literature^19^, the relative abundance of each homologous protein (*Z*_*i*_) was obtained^29^,^30^ (Eq. 2). With *Z*_*i*_, the weighted average protein length 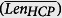, molecular weight 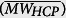, and amino acid content were calculated (Eqs 3, 4, 5). Step 2: the amino acid sequences of potentially N-glycosylated homologous proteins were analysed with the NetNGlyc 1.0 server^32^ to obtain the number of occupied N-linked sites per HCP 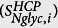. The weighted average number of N-linked glycosites across the CHO proteome 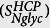 was obtained by combining 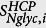 with *Z*_*i*_ (Eq. 6). Step 3: the CHO O-GalNAc glycoproteome^21^ was BLASTed^28^ against the SwissProt database^27^. The obtained list of homologous proteins was aligned with the one from the CHO proteome to obtain the weighted average number of O-GalNAc glycosites across the CHO proteome 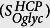 (Eq. 7). Step 4: the weighted average monosaccharide composition of CHO HCP N-linked 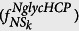 and O-GalNAc glycans 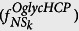 was obtained from the CHO glycome^20^ (Fig. 2) using Eqs 8 and 9. The average N-linked glycan monosaccharide composition for a commercial mAb 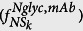 was also obtained from literature^23^ (Fig. 2). The demand of NSs towards glycolipid synthesis was obtained from the reported monosaccharide content of CHO cell glycosphingolipids relative to total protein content 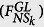
22. Step 5: the stoichiometric coefficients for the demand of NSs towards HCP N-linked 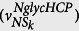, O-GalNAc 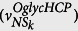, lipid 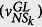, and mAb N-linked 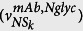 glycosylation were calculated using the corresponding equations included at the bottom of the figure using a dry cell weight (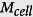) of 271pg/cell^34^, a protein content (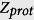) of 74.2% (w/w)^35^, and a mAb molecular weight (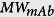) of 148,545 g/mol. All data and calculations are presented in Supplementary Tables ST1–ST10.

**Table 1:** mAb productivity to growth rate ratios and total protein synthesis rates.

	Productivity to growth ratios ( 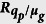 )					
	Low			High		
	Doolan *et al*.^24^	Chusainow *et al*.^25^	Kantardjieff *et al*.^26^	Doolan *et al*.^24^	Chusainow *et al*.^25^	Kantardjieff *et al*.^26^
*μ*_*g*_ (*h*^−1^)	0.031	0.03	0.025	0.011	0.011	0.011
*q*_*p*_ 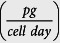	23.1	39.95	46.52	23.7	52.07	94.05
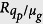 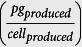	31.1	56.2	78.2	89.8	192	359.5
**Protein synthesis rates**						
	**Low**			**High**		
HCP 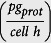	6.23	5.95	4.99	2.21	2.27	2.19
mAb 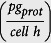	0.96	1.66	1.94	0.99	2.17	3.92
Total 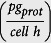	7.20	7.62	6.93	3.20	4.44	6.11
% to mAb	13.4%	21.9%	28.0%	30.9%	48.8%	64.1%

**Table 2:**
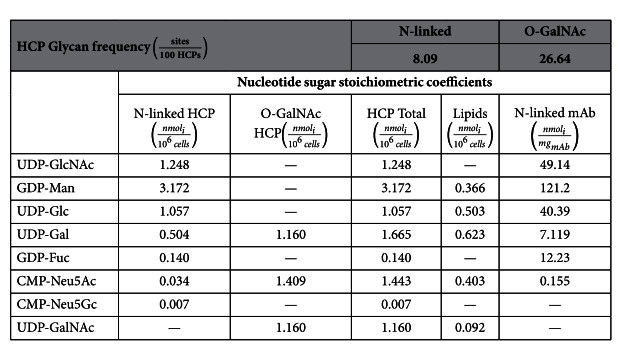
HCP glycan frequency and NS stoichiometric coefficients for CHO cell and mAb glycosylation.
